# Academic achievement is more closely associated with student-peer relationships than with student-parent relationships or student-teacher relationships

**DOI:** 10.3389/fpsyg.2023.1012701

**Published:** 2023-02-16

**Authors:** Xiaodan Yu, Xufei Wang, Haoyue Zheng, Xin Zhen, Min Shao, Haitao Wang, Xinlin Zhou

**Affiliations:** ^1^Department of Education, Ocean University of China, Qingdao, Shandong, China; ^2^State Key Laboratory of Cognitive Neuroscience and Learning, IDG/McGovern Institute for Brain Research, Siegler Center for Innovative Learning, Beijing Normal University, Beijing, China

**Keywords:** personal relationships, student-parent relationships, student-teacher relationships, student-peer relationships, academic achievement

## Abstract

**Introduction:**

Personal relationships have long been a concern in education. Most studies indicate that good personal relationships are generally positively correlated with academic performance. However, few studies have compared how different types of personal relationships correlate with academic performance, and the conclusions of existing studies are inconsistent. Based on a large sample, the current study compared how the three closest types of personal relationships among students (with parents, teachers, and their peers) compared with their academic performance.

**Methods:**

Cluster sampling was used to issue questionnaires to students in Qingdao City, Shandong Province, China in 2018 (Study 1) and in 2019 (Study 2). The actual sample size included 28168 students in Study 1 and 29869 students in Study 2 (both studies, Grades 4 and 8), thus totaling 58037 students. All students completed a personal relationship questionnaire and several academic tests.

**Results:**

The results showed that: (1) the quality of personal relationships significantly and positively correlated with academic performance; (2) Among the three types of relationships tested, the quality of student-peer relationships was the most closely associated with academic achievement.

**Discussion:**

This study gives insights into future research directions in this field and also reminds educators to pay attention to the personal relationships among their students, especially peer relationships.

## 1. Introduction

Numerous studies have shown a significant positive correlation between personal relationships and academic performance (Wentzel, 1998; Kiuru et al., 2009; Martin and Dowson, 2009; Tobbell and O'Donnell, 2013; Castro et al., 2015). In these studies, student relationships have typically been divided into those with parents (student-parent), those with teachers (student-teacher), and those with peers (student-peer) because these relationships encompass the main scope and characteristics of their daily activities (Rubin et al., 2010; Kiuru et al., 2014; Moore et al., 2018). Studies have shown that each of these relationship categories is an important factor in student development (Walsh et al., 2010; Wang and Eccles, 2012; King and Ganotice, 2014; Collie et al., 2016). Only a few studies have compared how well different types of personal relationships correlate with student academic performance (Chen, 2005; Lam et al., 2012; Altermatt, 2019; Wentzel et al., 2016; Leung et al., 2021; Vargas-Madriz and Konishi, 2021), and the results were not consistent. Which type of personal relationships is most closely related to academic performance remains unclear. We classified personal relationships the same way in the current study. With a large sample size (58,037 primary and middle school students) and two experiments (Experiment 1 and Experiment 2), the current study focused on the associations between student academic performance and relationships with family, teachers, and peers.

### 1.1. The correlation between personal relationships and academic achievement

Most studies agree that there is a significant correlation between the quality of personal relationships and academic performance; positive relationships can predict good academic performance, while those ridden with conflict can predict poor academic performance. These studies focused on the effects of different types of personal relationships on academic performance.

First, considering the student-parent relationships, studies have found that parental support is a predictor of student achievement (Wentzel, 1998; Chiu, 2010; Castro et al., 2015; Igbo et al., 2015). For example, Igbo et al. (2015) used an Ex-Post Facto design to examined 48 public schools in the Otukpo Education Zone of Benue State and formulated and tested four null hypotheses with the t-test statistics at 0.05 level of significance. They found that student-parent relationships had a significant impact on math and English test scores. Castro et al. (2015) found that the frequency with which students interact with their family members had a positive impact on academic performance. Based on this, the authors concluded that parents influence students by shaping the positive value of education in academic-oriented behavior.

Second, the student-teacher relationships have also been shown impact academic performance (Roorda et al., 2011; Hughes et al., 2012; Ly et al., 2012; Hajovsky et al., 2020). High quality student-teacher relationships could contribute to students’ cognitive skill development. For example, when Ly et al. (2012) characterized student-teacher relationships as either intimacy, conflict, or warmth, they found that the comprehensive warmth score (rated by the teachers) was significantly and positively correlated with children’s math and reading performance. Hajovsky et al. (2020) studied two types of student-teacher relationships, intimacy and conflict, and found that while intimacy (as rated by the teachers) had only an indirect effect on math achievement, conflict affected math achievement both directly and indirectly.

Third, student-peer relationships have been shown to be significantly associated with student academic performance (Espelage et al., 2013; Li et al., 2020; Vignery and Laurier, 2020). For example, using both horizontal and longitudinal analysis, Espelage et al. (2013) showed that bullying and peer victimization were related to low academic performance. In the study of Gremmen et al. (2018), a longitudinal social network analysis (RSiena) showed that student academic engagement and achievement improved when their friends scored better, and vice versa, regardless of their physical position in the classroom. The selection effect (i.e., students for groups of friends that share the same levels of academic performance) and socialization process (i.e., peer performance is significantly associated with future student achievement), which were reported by Vignery and Laurier (2020), could be used to understand the links between student academic performance and peer relationships.

Despite this evidence, other studies have come to different conclusions. These have suggested that there is no significant correlation between personal relationships and academic achievement (Chen, 2008; Nokali et al., 2010; Barile et al., 2012; Hajovskya et al., 2017). For example, Chen (2008) found perceived peer support had no significant direct or indirect relationship with student achievement at any grade level, while perceived academic engagement was significantly and positively related with student achievement for adolescents at all grade levels. Nokali et al. (2010) found that improvements in parental involvement predicted declines in problematic behaviors and improvements in social skills, but did not predict changes in achievement. Barile et al. (2012) found that even if students have good relationships with their teachers, the relationship may not be enough to improve their math achievement, particularly in high school. Hajovskya et al. (2017) found that student-teacher conflict had a statistically significant effect on subsequent math performance (*β* = 0.04), but the size of the effect was not actually significant. The connection between personal relationships and academic achievement remains unclear and more research is needed to reach a definitive conclusion.

### 1.2. Comparative study of the association between different types of personal relationships and academic achievement in students

Determining which type of personal relationships is most closely related to academic performance will contribute to future theoretical and practical education planning. We found only a few studies that have compared how well different types of personal relationships correlate with student academic performance (Chen, 2005; Lam et al., 2012; Altermatt, 2019; Wentzel et al., 2016; Leung et al., 2021; Vargas-Madriz and Konishi, 2021). Importantly, among the existing research, results were not consistent.

Some studies state more specifically which type of personal relationships is important. For instance, Altermatt (2019) found that only perceived academic support from peers, neither parents nor professors, independently predicted academic self-efficacy. Gao and Xue (2020) found that parental participation had a greater impact on student academic performance than did peer influence, and was better at explaining differences in student academic performance. Vargas-Madriz and Konishi (2021) found that parental support showed a direct relationship with academic involvement, while the relationships between peer/teacher support and academic involvement was mediated by the students’ sense of school belongingness.

Other studies found some personal relationships have less correlation with academic performance than other personal relationships. Some studies found that student-parent and student-peer relationships were more related to academic achievement than were student-teacher relationships. For instance, Leung et al. (2021) reported that while the quality of student-parent and student-peer relationships at Time 1 were significantly associated with academic achievement at Time 2 after controlling for Time 1 academic achievement, that of student-teacher relationships was not. In contrast, other studies found that student-parent and student-teacher relationships more related to academic achievement than were student-peer relationships. For instance, Wentzel et al. (2016) examined perceived emotional support and expectations from parents, teachers, and classmates in relation social behavior and academic functioning in Mexican American adolescents (*n* = 398). Results of a regression analyses indicated that teacher and parent variables were significant predictors of academic outcomes, while peer variables were significant predictors of social behavior. Lam et al. (2012) examined multiple-group structural equation modeling to revealed that, not perceptions of peer support, but teacher support and parent support were related indirectly to academic performance through student engagement.

Thus, the literature shows varying results regarding the correlation between different types of student relationships and student academic performance. There are several reasons that could explain why these results have been inconsistent.

First, the samples in these studies had differences in age, size, and social and cultural backgrounds. For example, consider the following two studies. In Altermatt (2019), the participants were 107 undergraduate students (79 women, 28 men), who were of traditional college age (mean = 20.14 years) and the majority (92%) identified as Caucasian, enrolled in introductory psychology courses at a liberal arts college in the Midwest United States. In contrast, Leung et al. (2021) conducted a longitudinal study in Hong Kong, China in which data were collected in two waves from a sample of 786 primary school students who had similar socioeconomic status and academic performance.

In addition, the disciplines chosen in the literature to test academic performance varied considerably. For instance, Teng et al. (2018) used final exam results in Chinese, mathematics, and English from the semester just before the experiment. The scores of each participant were standardized within every class, which yielded Z-scores for the three subjects. The sum of the three Z scores was used as an index of each student’s academic achievement. In contrast, García Bacete et al. (2021) used student marks in mathematics and Spanish at the end-of-year exams for 1st, 2nd, 4th, and 6th grades, applying a 5-point scale (fail, pass, good, very good, and excellent).

Based on these factors that might influence the research results, the current study makes adjustments in a large sample, which can ameliorate the limitations of previous studies. We focused on comparing three student relationships using different disciplines in two studies. This method allows us to determine which relationship is most closely related to academic achievement.

### 1.3. How the association between personal relationships and academic achievement develops

Previous research has focused on whether the correlation between personal relationships and academic achievement varies by grade. Most researchers have found that the degree to which personal relationships affect academic achievement varies by grade. For example, Furman and Buhrmester (1992) observed age differences in perceptions of relationships with parents, grandparents, teachers, and siblings. Parents were seen as the most frequent providers of support in the fourth grade, same-sex friends were perceived to be as supportive as parents in the seventh grade, and were the most frequent providers of support in the tenth grade. Another good example of this can be found in Gallardo’ research (2016). Their regression analysis indicated that peer acceptance positively predicted subsequent academic achievement and this relationship was also moderated by age, with the effect of peer acceptance on subsequent academic achievement being weaker during mid-adolescence than in early adolescence. This consistent result was also present in Wentzel’ study. Wentzel et al. (2020) found an association between peer social acceptance and academic performance as twice as strong for students of primary education with respect to students of secondary education.

These findings indicate that various relationships are perceived as playing different roles at different points in development, which is consistent with physical and mental development in children. Due to the development of self-awareness and independence, children and teenagers show completely different personal communication characteristics; the focus of their personal relationships gradually shift to close friendships. Therefore, we have reason to believe that with this change in focus, the correlation between personal relationships and academic performance also changes.

### 1.4. Current study

Based on the existing theoretical and empirical results, this study used a big sample to focus on three different personal relationships among students and their academic performance. The data came from monitoring education quality in the Chinese city of Qingdao (Shandong Province) for two consecutive years, which includes a survey that assesses personal relationships in fourth and eighth graders. Part of the personal relationship scale was updated in the second year (see Measurements in Study 2 for details). As indexes of academic achievement, we chose math scores in the first year and scientific scores in the second year.

Social Impact Theory (SIT) provides theoretical support for our hypothesis. The SIT proposed by Latan (1981) suggests that an individual’s feelings, attitudes, and behavior can be influenced by the presence of others. When some numbers of social sources are acting on a target individual, the amount of impact experienced by the target should be a multiplicative function of the strength, S, the immediacy, I, and the number, N, of sources present. The three aspects of SIT is to say that how important the group of social sources is to oneself (strength of influence), closeness to the group (in proximity), and size of the group (numbers) all combine to influence individuals. Pedersen et al. (2008) proved the principles of SIT may contribute to differences between assessments performed individually and those completed when surrounded by members of one’s salient reference group. They examined 284 members of campus organizations in two contexts (online and group) to determine if individuals endorse different responses to questions of perceived and actual drinking norms across contexts. Results showed that all participants endorsed higher responses on questions of actual and perceived group behavior and of perceived group attitudes toward drinking during the group assessment than online assessment.

From this perspective, the characteristics of peer relationships are consistent with the important factors that influence individuals as emphasized in SIT theory. Usually, a class size is around 30 to 50 students. School-aged children spend most of their time in school and have more contact with their classmates and friends. The number of peers and communication time with peers are far greater than that of parents and teachers. A Chinese classic is consistent with this saying, “If you stay close to vermilion, you will be red, and if you stay close to ink, you will be black.” (from Fu Xuan, Jin Dynasty, China), which addressed the importance of immediacy.

An equal relationship with peers can provide a sense of psychological stability and identity, as well as opportunities and places to use their own initiative, which parents and teachers cannot provide. Students can meet their self-development needs through mutual help and respect by sharing common feelings, conflicts, worries and difficulties with friends.

In addition, the process through which children acquire knowledge is not done alone, but requires communication and experience. With improved understanding of classroom teaching, cooperative learning has become a focus in education and many studies have confirmed the positive effects it has on different outcomes (Kyndt et al., 2013). It is expected that with the strong advocacy of cooperative learning in recent years, students are more inclined to cooperate with their peers to complete their learning tasks. Such a reform further promotes the degree of intimacy between peers and the importance of peers in student learning.

To sum up, we hypothesized that among the three typical personal relationships (student-parent, student-teacher, and student-peer), the student-peer relationships would have the highest correlation with academic achievement.

## 2. Study 1

### 2.1. Methods

#### 2.1.1. Participants

With the help of the center of Assessment for Basic Education Quality in Qingdao City, Shandong Province, China, we used the cluster-sampling method to send questionnaires to each school. The data were collected in May 2018, and included 28,726 students in Grades 4 and 8 who came from 489 primary schools and 238 middle schools. Excluding 558 students who incorrectly filled out the forms (erroneous gender, grade level, or age), the actual sample size was 28,168. Parental consent was obtained prior to classroom-based testing.

After excluding invalid questionnaires, 17,112 participants (mean age = 9.80 years) remained in Grade 4, which included 8,847 (51.7%) boys and 8,265 (48.3%) girls, and 11,056 participants (mean age = 13.65 years) remained in Grade 8, which included 5,448 (49.28%) boys and 5,608 (50.72%) girls. Detailed demographic information is shown in Table 1.

**Table 1:** Sample size and mean age (years) by gender, grade level, and math test version.

	Grade 4			Grade 8	
	Number	Mean age		Number	Mean age
	(Male, Female)	(Male, Female)		(Male, Female)	(Male, Female)
Test 1	2,859	9.79	Test 1	1840	13.66
	(1,458, 1,401)	(9.81, 9.77)		(947, 893)	(13.69, 13.64)
Test 2	2,852	9.79	Test 2	1847	13.64
	(1,429, 1,423)	(9.79, 9.80)		(901, 946)	(13.66, 13.61)
Test 3	2,856	9.79	Test 3	1832	13.66
	(1,463, 1,393)	(9.79, 9.80)		(906, 926)	(13.68, 13.63)
Test 4	2,854	9.79	Test 4	1843	13.66
	(1,488, 1,366)	(9.80, 9.78)		(884, 959)	(13.70, 13.62)
Test 5	2,852	9.80	Test 5	1850	13.66
	(1,488, 1,364)	(9.81, 9.80)		(891, 959)	(13.69, 13.63)
Test 6	2,839	9.81	Test 6	1844	13.64
	(1,521, 1,318)	(9.82, 9.79)		(919, 925)	(13.70, 13.59)
Total	17,112	9.80	Total	11,056	13.65
	(8,847, 8,265)	(9.80, 9.79)		(5,448, 5,608)	(13.69, 13.62)

We divided the math questions for each grade into six sets of tests in which the specific content differed, but the combination of questions was the same.

#### 2.1.2. Measurements

Personal relationships were measured using a revised version of the Personal Relationship Assessment Scale (PRAS) developed by the center of Qingdao Education Evaluation and Quality Monitoring Center, Ocean University of China. PRAS comprises 10 items that focus on perceived personal interaction of students with parents, teachers, and their peers (e.g., “When I’m in trouble, teacher help me in time”).Students responded to each item about their particular situations on a 5-point Likert scale ranging from 1 (*strongly disagree*) to 5 (*strongly agree*). Scores were summed across the 10 questions to generate the final score with higher scores indicating higher quality of personal relationships students perceived. The scale has good internal consistency, with Cronbach’s alpha of 0.648 for Grade 4 and 0.763 for Grade 8.

The math achievement test was designed based on the mathematic curriculum standards of China, and included single choice, fill-in-the-blank, and essay problems that test math knowledge for Grades 4 and 8. For the sake of scientific research, we divided the test into six sets of test papers with different content and the same question type combination, which were randomly distributed to students in each grade. Test papers for each grade (6 sets for each grade) had good internal consistency. Cronbach’s alpha values for the 6 sets of Grade 4 and Grade 8 papers ranged from 0.720 to 0.808.

#### 2.1.3. Procedure

After obtaining the informed consent of teachers and students, we began data collection in 2018, requiring all participants to complete the PRAS and a random math test corresponding to their grade. The math scores were graded by a quality inspection team of professionals. Moreover, the quality inspection team was also responsible for processing the questionnaire data. Questionnaire answers and math test results were returned to the researchers in excel format.

All tasks were conducted in a school environment and started at the same time. Relationships were measured online with no time limit, and all students received the same instructions. The math tests were completed in written form with a 60-min time limit.

#### 2.1.4. Data analysis

Before analysis, we cleaned up the data using Microsoft Visual FoxPro 9.0. This was mainly to replace variable names, delete outliers, null values, and add dimensions. The purpose was to ensure that the data being analyzed tended to be true and accurate.

The data analyses were conducted with IBM SPSS Statistics for Windows, Version 26.0. Reliability and validity analysis, descriptive analysis, and inferential analyses were performed to examine the distribution of all variables and the associations between the main variables and other variables. The inferential analysis used Pearson correlation analysis and repeated-measures one-way analysis of variance (ANOVA) to explore the association between personal relationships and math achievement, as well as gender and age differences among them.

### 2.2. Results

#### 2.2.1. Mean values and gender differences in personal relationships

Table 2 shows mean values and gender differences in overall personal relationships and the three subdivisions. There is a significant difference in gender for students’ personal relationships that PRAS scores were higher for girls than for boys in both Grade 4 [girls vs. boys: 4.34 vs. 4.14, *t*(*df*) = −19.341 (17098.681), *p* < 0.001] and Grade 8 [4.41 vs. 4.26, *t*(*df*) = −11.298 (10936.739), *p* < 0.001]. Looking at individual relationship types, the same pattern held for the student-parent [Grade 4: 4.16 vs. 4.04, *t*(*df*) = −11.111 (17095.336), *p* < 0.001, Grade 8: 4.23 vs. 4.15, *t*(*df*) = −5.711 (11054), *p* < 0.001]; student-teacher, Grade 4: 4.68 vs. 4.57, *t*(*df*) = −9.322 (16976.821), *p* < 0.001, Grade 8: 4.55 vs. 4.51, *t*(*df*) = −2.878 (10860.621), *p* < 0.01 and student-peer, Grade 4: 4.05 vs. 3.68, *t*(*df*) = −22.937 (17106.491), *p* < 0.001, Grade 8: 4.35 vs. 4.02, *t*(*df*) = −19.179 (10637.617), *p* < 0.001.

**Table 2:** Mean (SD) values and independent sample T test results for gender in personal relationships.

Relationship		Grade 4				Grade 8			
		Boys	Girls	*t*	*p*	Boys	Girls	*t*	*p*
		*M(SD)*	*M(SD)*			*M(SD)*	*M(SD)*		
Student-parent	Test 1	4.05 (0.74)	4.16 (0.71)	−4.010	<0.001	4.13 (0.79)	4.23 (0.75)	−2.837	0.005
	Test 2	4.03 (0.70)	4.14 (0.70)	−4.171	<0.001	4.19 (0.76)	4.25 (0.78)	−1.509	0.131
	Test 3	4.06 (0.73)	4.15 (0.68)	−3.262	0.001	4.10 (0.79)	4.21 (0.74)	−3.141	0.002
	Test 4	4.01 (0.72)	4.17 (0.67)	−6.322	<0.001	4.12 (0.82)	4.25 (0.77)	−3.348	0.001
	Test 5	4.04 (0.70)	4.17 (0.69)	−5.177	<0.001	4.19 (0.75)	4.20 (0.74)	−0.373	0.709
	Test 6	4.04 (0.74)	4.16 (0.70)	−4.325	<0.001	4.15 (0.81)	4.24 (0.75)	−2.658	0.008
	Total	4.04 (0.72)	4.16 (0.69)	−11.111	<0.001	4.15 (0.79)	4.23 (0.75)	−5.711	<0.001
Student-teacher	Test 1	4.58 (0.81)	4.69 (0.69)	−3.686	<0.001	4.51 (0.89)	4.55 (0.80)	−1.201	0.230
	Test 2	4.57 (0.82)	4.69 (0.70)	−4.263	<0.001	4.55 (0.85)	4.56 (0.82)	−0.337	0.736
	Test 3	4.58 (0.79)	4.68 (0.70)	−3.358	0.001	4.47 (0.86)	4.55 (0.75)	−2.184	0.029
	Test 4	4.57 (0.82)	4.67 (0.70)	−3.794	<0.001	4.47 (0.91)	4.59 (0.77)	−3.013	0.003
	Test 5	4.55 (0.83)	4.68 (0.71)	−4.364	<0.001	4.53 (0.83)	4.52 (0.78)	0.227	0.821
	Test 6	4.58 (0.83)	4.67 (0.70)	−3.324	0.001	4.52 (0.88)	4.54 (0.78)	−0.499	0.618
	Total	4.57 (0.82)	4.68 (0.70)	−9.322	<0.001	4.51 (0.87)	4.55 (0.79)	−2.878	0.004
Student-peer	Test 1	3.71 (1.09)	4.06 (1.01)	−8.811	<0.001	4.01 (0.99)	4.35 (0.83)	−7.851	<0.001
	Test 2	3.62 (1.12)	4.04 (1.02)	−10.379	<0.001	4.05 (0.99)	4.40 (0.81)	−8.308	<0.001
	Test 3	3.69 (1.11)	4.08 (0.99)	−9.888	<0.001	3.97 (0.98)	4.32 (0.85)	−8.285	<0.001
	Test 4	3.64 (1.10)	4.01 (1.03)	−9.298	<0.001	4.01 (0.99)	4.35 (0.83)	−7.881	<0.001
	Test 5	3.66 (1.11)	4.03 (1.03)	−9.122	<0.001	4.07 (0.95)	4.31 (0.85)	−5.673	<0.001
	Test 6	3.73 (1.10)	4.08 (1.02)	−8.737	<0.001	4.00 (0.99)	4.37 (0.80)	−8.954	<0.001
	Total	3.68 (1.11)	4.05 (1.02)	−22.937	<0.001	4.02 (0.98)	4.35 (0.83)	−19.179	<0.001
Total	Test 1	4.16 (0.72)	4.33 (0.67)	−6.588	<0.001	4.25 (0.77)	4.41 (0.69)	−4.724	<0.001
	Test 2	4.12 (0.71)	4.33 (0.67)	−8.300	<0.001	4.31 (0.73)	4.45 (0.71)	−4.082	<0.001
	Test 3	4.15 (0.70)	4.35 (0.68)	−7.521	<0.001	4.20 (0.77)	4.39 (0.69)	−5.737	<0.001
	Test 4	4.12 (0.70)	4.33 (0.68)	−8.167	<0.001	4.24 (0.76)	4.45 (0.68)	−6.273	<0.001
	Test 5	4.12 (0.71)	4.34 (0.67)	−8.586	<0.001	4.31 (0.71)	4.39 (0.70)	−2.412	0.016
	Test 6	4.15 (0.71)	4.36 (0.68)	−8.240	<0.001	4.26 (0.75)	4.40 (0.69)	−4.360	<0.001
	Total	4.14 (0.71)	4.34 (0.68)	−19.341	<0.001	4.26 (0.75)	4.41 (0.70)	−11.298	<0.001

*p < 0.05, **p < 0.01, ***p < 0.001.

#### 2.2.2. The correlation analysis between personal relationships and math achievement

PRAS scores correlated significantly and positively with math achievement in Grade 4 (*r* = 0.246, *p* < 0.01) and Grade 8 (*r* = 0.191, *p* < 0.01). To further explore this relationship, we analyzed the data according to the test version, gender, and grade. We found that the correlation between personal relationships and math achievement was roughly consistent and stable. See Table 3 for details.

Student-parent relationships. For boys in Grade 4, the correlation coefficient between the student-parent PRAS score and math achievement overall was 0.182 (*p* < 0.01). When looking each math test version separately, the correlation coefficients ranged from 0.134 to 0.221 (all *p* < 0.01). For girls in Grade 4, the correlation coefficient for the same relationship was 0.189 overall (*p* < 0.01), and ranged from 0.153 to 0.254 (all *p* < 0.01) for each math test individually. Looking at the same relationship in Grade 8, the correlation coefficient was 0.154 for boys (*p* < 0.01; ranging from 0.097 to 0.197, all *p* < 0.01) and 0.114 for girls (*p* < 0.01; ranging from 0.078 to 0.156, *p* < 0.05).

**Table 3:** Correlations between personal relationships and math scores.

Relationship		Grade 4			Grade 8		
		Boys	Girls	Total	Boys	Girls	Total
Student-parent	Test 1	0.134**	0.254**	0.187**	0.191**	0.156**	0.176**
	Test 2	0.218**	0.221**	0.220**	0.147**	0.112**	0.129**
	Test 3	0.189**	0.209**	0.199**	0.197**	0.104**	0.153**
	Test 4	0.178**	0.220**	0.195**	0.097**	0.078*	0.090**
	Test 5	0.221**	0.153**	0.188**	0.149**	0.128**	0.138**
	Test 6	0.192**	0.196**	0.190**	0.171**	0.150**	0.161**
	Total	0.182**	0.189**	0.185**	0.154**	0.114**	0.136**
Student-teacher	Test 1	0.145**	0.160**	0.149**	0.170**	0.127**	0.152**
	Test 2	0.171**	0.215**	0.191**	0.130**	0.136**	0.133**
	Test 3	0.135**	0.172**	0.153**	0.142**	0.143**	0.142**
	Test 4	0.112**	0.133**	0.120**	0.102**	0.073*	0.091**
	Test 5	0.188**	0.124**	0.160**	0.158**	0.165**	0.161**
	Test 6	0.145**	0.158**	0.148**	0.135**	0.126**	0.130**
	Total	0.141**	0.151**	0.145**	0.135*	0.122**	0.129**
Student-peer	Test 1	0.206**	0.309**	0.245**	0.197**	0.187**	0.194**
	Test 2	0.237**	0.262**	0.247**	0.223**	0.199**	0.211**
	Test 3	0.278**	0.244**	0.265**	0.253**	0.199**	0.225**
	Test 4	0.247**	0.279**	0.257**	0.209**	0.164**	0.191**
	Test 5	0.301**	0.292**	0.291**	0.224**	0.240**	0.229**
	Test 6	0.261**	0.221**	0.234**	0.198**	0.206**	0.197**
	Total	0.239**	0.250**	0.241**	0.212**	0.188**	0.200**
Totals	Test 1	0.215**	0.329**	0.262**	0.230**	0.180**	0.209**
	Test 2	0.271**	0.296**	0.282**	0.200**	0.196**	0.199**
	Test 3	0.252**	0.258**	0.256**	0.234**	0.170**	0.203**
	Test 4	0.233**	0.263**	0.243**	0.165**	0.113**	0.144**
	Test 5	0.300**	0.254**	0.275**	0.216**	0.208**	0.212**
	Test 6	0.267**	0.252**	0.252**	0.223**	0.196**	0.209**
	Total	0.242**	0.256**	0.246**	0.208**	0.170**	0.191**

*p < 0.05, **p < 0.01.

In Grade 4, the correlation coefficient between student-teacher PRAS score and math achievement was 0.141 for boys (*p* < 0.01; ranging from 0.112 to 0.188 for the six math tests, all *p* < 0.01) and 0.151 for girls (*p* < 0.01; ranging from 0.124 to 0.215, *p* < 0.01). In Grade 8, the correlation coefficient was 0.135 for boys (*p* < 0.05; ranging from 0.102 to 0.170, all *p* < 0.01) and 0.122 for girls (*p* < 0.01; ranging from 0.073 to 0.165, all *p* < 0.05).

In Grade 4, the correlation coefficient between student-peer PRAS score and math achievement was 0.239 for boys (*p* < 0.01; ranging from 0.206 to 0.301, all *p* < 0.01) and 0.250 for girls (*p* < 0.01; ranging from 0.221 to 0.309, all *p* < 0.01). In Grade 8, the correlation coefficient was 0.212 for boys (*p* < 0.01; ranging from 0.197 to 0.253, all *p* < 0.01) and 0.188 for girls (*p* < 0.01; ranging from 0.164 to 0.240, all *p* < 0.01).

In Grade 4, the correlation coefficient between the overall PRAS score and math achievement was 0.242 for boys (*p* < 0.01; ranging from 0.215 to 0.300, all *p* < 0.01) and 0.256 for girls (*p* < 0.01; ranging from 0.252 to 0.329, all *p* < 0.01). In Grade 8, it was 0.208 for boys (*p* < 0.01; ranging from 0.165 to 0.234, all *p* < 0.01) and 0.170 for girls (*p* < 0.01; ranging from 0.113 to 0.208, all *p* < 0.01).

Based on these results, we analyzed the data using a repeated-measures General Linear Model. The in-between factors were the correlation coefficients between the three types of student relationships and math achievement. The factors were Grade (4 or 8) and gender, and the Sphericity Assumption method was adopted to obtain the results of the intra-group Factor test (see Table 4). We found significant differences between the correlations (*p* < 0.001). Based on Table 3, we can see that among the three relationship types, the student-peer relationships had the closest association with math achievement.

**Table 4:** Results of intra-group factor test.

Source	SS	*df*	*MS*	*F*	*P*
Personal relationships	0.105	2	0.052	95.386	<0.001
Gender	0.000	2	0.000	0.208	0.813
Grade	0.005	2	0.003	4.558	0.016
Gender × Grade	0.001	2	0.001	0.924	0.405
Error (personal relationships)	0.022	40	0.001		

*p < 0.05, **p < 0.01, ***p < 0.001, Bonferroni-corrected.

#### 2.2.3. Multivariate ANOVA

Multi-factor ANOVAs were used to investigate gender and grade differences in the correlation between student personal relationships and math achievement. We found no gender difference for any of the three relationship types (all *p* > 0.05, Bonferroni-corrected). In contrast, we did find a difference in grade for the student-parent [*F*(1, 20) =19.134, *p* < 0.001, Bonferroni-corrected, *η^2^ p* = 0.489] and student-peer [*F*(1, 20) = 21.236, *p* < 0.001, Bonferroni-corrected, *η^2^ p* = 0.515] relationships. Combined with Tables 3, 5, we conclude that the correlation between the quality of the student-peer relationships and math achievement was stronger in Grade 4 than in Grade 8, and it was same between student-parent relationships and math achievement. See Table 5 for details.

**Table 5:** ANOVA for gender and grade in relation to personal relationships and achievement.

	Student-parent relationship				Student-teacher relationship				Student-peer relationship			
	*F*	*df*	*p*	*η^2^ p*	*F*	*df*	*p*	*η^2^ p*	*F*	*df*	*p*	*η^2^ p*
Gender	0.408	1	0.530	0.020	0.000	1	0.994	0.000	0.053	1	0.820	0.003
Grade	19.134	1	<0.001	0.489	3.225	1	0.088	0.139	21.236	1	<0.001	0.515
Gender × Grade	4.582	1	0.045	0.186	0.905	1	0.353	0.043	1.805	1	0.194	0.083
Error		20				20				20		

*p < 0.05, **p < 0.01, ***p < 0.001, Bonferroni-corrected.

### 2.3. Discussion

The results of Study 1 supported our hypothesis that student-peer relationships have the closest association with academic achievement (as indexed by math achievement) among the three types of student relationships.

The correlation analysis showed that personal relationships significantly and positively correlate with math performance, which is consistent with numerous other studies (Dhingra and Manhas, 2009; Lee, 2012; Oberle and Schonert-Reichl, 2013). Further analysis showed that, among the three types of student relationships, the student-peer relationships had the closest association with math scores in both Grade 4 and Grade 8, among boys and girls. To the best of our knowledge, this study is the first evidence showing that the student-peer relationships have a closer association with academic achievement than do student-parent or student-teacher relationships.

The ANOVA results showed the correlation between student-peer relationships quality and math performance depended on grade: the correlation became less as students got older. In terms of changes in peer relationships themselves, it has been confirmed that increased cognitive ability may encourage older students to seek independence more than younger students do (Furman and Buhrmester, 1992; Fuligni Andrew and Eccles Jacquelynne, 1993). Therefore, we speculate that eighth grade students may be more mature in thinking and more independent in their learning compared with fourth grade students, which could explain the grade difference.

Further inspiration comes from Chen (2005). Chen tested a hypothesized model that students’ self-perceived academic support (from parents, teachers, and peers) is directly or indirectly related to their achievement through their own perceived academic engagement. In this process, the author found that personal relationships have different effects on different academic subjects: student-teacher relationships had the greatest impact on English and math, while student-peer relationships had the least impact. The student-teacher relationships had the greatest influence on Chinese, and the student-parent relationships had the least influence. These findings are not consistent with our results from Study 1, and we are curious whether differences in discipline is the key factor leading to different results. Therefore, in the second year, we focused on the connection between personal relationships and science achievement to test our hypothesis that among the three typical personal relationships (student-parent, student-teacher, and student-peer), the student-peer relationships would have the highest correlation with academic achievement.

## 3. Study 2

### 3.1. Method

#### 3.1.1. Participants

We used the same methods to collect student data as in Study 1. The data were collected in May 2019, and included 30,596 students in Grade 4 and Grade 8 who came from 545 primary schools and 241 middle schools. Excluding 727 students who incorrectly filled out the forms (erroneous gender, grade level, or age), the actual sample size was 29,869. After excluding invalid questionnaires, there were 17,752 participants (mean age = 9.80 years) in Grade 4, which included 9,210 (51.9%) boys and 8,542 (48.1%) girls, and 12,117 participants (mean age = 13.75 years) in Grade 8, which included 6,283 (51.9%) boys and 5,834 (48.1%) girls. Parental consent was obtained prior to classroom-based testing. Detailed demographic information is shown in Table 6.

**Table 6:** Sample size and mean age (years) by gender, grade level, and science test version.

	Grade 4			Grade 8	
	Number	Mean age		Number	Mean age
	(Male, Female)	(Male, Female)		(Male, Female)	(Male, Female)
Test 1	4,482	9.81	Test 1	1,836	13.74
	(2,320, 2,162)	(9.82, 9.79)		(945, 891)	(13.74, 13.74)
Test 2	4,460	9.79	Test 2	2,065	13.75
	(2,328, 2,132)	(9.79, 9.80)		(1,071, 994)	(13.75, 13.75)
Test 3	4,418	9.80	Test 3	2,059	13.76
	(2,330, 2088)	(9.80, 9.80)		(1,033, 1,026)	(13.79, 13.72)
Test 4	4,392	9.80	Test 4	2,056	13.76
	(2,232, 2,160)	(9.81, 9.78)		(1,069, 987)	(13.80, 13.72)
			Test 5	2,052	13.75
				(1,076, 976)	(13.77, 13.73)
			Test 6	2,049	13.74
				(1,089, 960)	(13.76, 13.73)
Total	17,752	9.80	Total	12,117	13.75
	(9,210, 8,542)	(9.80, 9.79)		(6,283, 5,834)	(13.77, 13.73)

We divided the science questions for Grade 4 into four sets, and the science knowledge for Grade 8 into six sets, which the specific content differed, but the combination of questions was the same.

#### 3.1.2. Measurements

Personal relationships were measured using PRAS (2019), an updated version of PRAS (2018) that still focus on perceived personal interaction of students with parents, teachers, and their peers (e.g., “I have good times with my classmates”). Since large-scale educational quality monitoring generally requires a certain amount of annual updates, PRAS (2019) has been revised based on last year’s version: the student-parent section was revised from four items, adding reverse scoring questions to eight items; a reverse scoring question was added to the student-teacher section and a forward scoring question was added to the student-peer section. The scoring rules remain the same. Students responded to each of 17 items about their particular situations. The scale has good internal consistency, with Cronbach’s alphas of 0.869 for Grade 4 and 0.916 for Grade 8.

The Science achievement test was designed based on the science curriculum standards of China, which include single choice questions and non-choice questions and content about science knowledge in Grade 4 and Grade 8. For the experiment, we divided the Grade 4 test into four sets and Grade 8 test into six sets, each with different content but with the same combination of questions, which were randomly distributed to students in each grade. Test papers for each grade had good internal consistency, with Cronbach’s alpha values for the 4 sets of test papers in Grade 4 and Grade 8 ranging from 0.723 to 0.922.

#### 3.1.3. Procedure

After obtaining the informed consent of teachers and students, we began data collection in 2019, requiring all participants to complete the PRAS and a random science test corresponding to their grade. The science scores were graded by a quality inspection team of professionals. All other aspects of the procedure were the same as in Study 1.

#### 3.1.4. Data analysis

Data analysis was conducted in the same manner as in Study 1.

### 3.2. Results

#### 3.2.1. Mean values and gender differences in personal relationships

Table 7 shows mean values and gender differences in overall personal relationships and the three subdivisions. There is a significant difference in gender for students’ personal relationships that PRAS scores were higher for girls than for boys in both Grade 4 [girls vs. boys: 4.40 vs. 4.25, *t*(*df*) = 12.940 (17747.529), *p* < 0.001] and Grade 8 [4.16 vs. 4.07, *t*(*df*) = 5.854 (12115), *p* < 0.001]. Looking at individual relationship types, the same pattern held for the student-parent, Grade 4: 4.31 vs. 4.20, *t*(*df*) = 7.817 (17629.440), *p* < 0.001, Grade 8: 3.95 vs. 3.92, *t*(*df*) = 1.548 (12115), *p* = 0.122; student-teacher, Grade 4: 4.65 vs. 4.50, *t*(*df*) = 12.694 (17545.281), *p* < 0.001, Grade 8: 4.38 vs. 4.28, *t*(*df*) = 5.827 (12107.187), *p* < 0.001 and student-peer, Grade 4: 4.39 vs. 4.21, *t*(*df*) = 13.822 (17749.549), *p* < 0.001, Grade 8: 4.43 vs. 4.28, *t*(*df*) = 9.844 (12113.495), *p* < 0.001.

**Table 7:** Mean (SD) values and independent sample T test results for gender in personal relationships.

Relationship		Grade 4					Grade 8			
		Boys	Girls	*t*	*p*		Boys	Girls	*t*	*p*
		*M(SD)*	*M(SD)*				*M(SD)*	*M(SD)*		
Student-parent	Test 1	4.20 (0.86)	4.32 (0.86)	4.663	<0.001	Test 1	3.93 (1.02)	3.99 (0.97)	1.282	0.200
	Test 2	4.25 (0.84)	4.30 (0.86)	2.097	0.036	Test 2	3.90 (1.00)	4.00 (1.00)	2.235	0.026
	Test 3	4.18 (0.86)	4.31 (0.84)	5.237	<0.001	Test 3	3.88 (1.01)	3.93 (1.01)	1.074	0.283
	Test 4	4.19 (0.86)	4.29 (0.88)	3.649	<0.001	Test 4	3.91 (1.02)	3.90 (1.02)	−0.225	0.822
						Test 5	3.92 (1.01)	3.92 (1.03)	0.048	0.962
						Test 6	3.96 (0.97)	3.94 (1.04)	−0.516	0.606
Student-teacher	Total	4.20 (0.86)	4.31 (0.86)	7.817	<0.001	Total	3.92 (1.00)	3.95 (1.01)	1.548	0.122
	Test 1	4.50 (0.84)	4.65 (0.69)	6.67	<0.001	Test 1	4.25 (0.96)	4.36 (0.90)	2.582	0.010
	Test 2	4.52 (0.82)	4.65 (0.70)	5.594	<0.001	Test 2	4.28 (0.96)	4.40 (0.87)	3.206	0.001
	Test 3	4.48 (0.85)	4.65 (0.68)	7.472	<0.001	Test 3	4.29 (0.95)	4.33 (0.90)	1.048	0.295
	Test 4	4.51 (0.82)	4.64 (0.70)	5.638	<0.001	Test 4	4.30 (0.94)	4.36 (0.87)	1.580	0.114
						Test 5	4.28 (0.96)	4.39 (0.84)	2.959	0.003
						Test 6	4.31 (0.96)	4.43 (0.82)	3.060	0.002
Student-peer	Total	4.50 (0.83)	4.65 (0.69)	12.694	<0.001	Total	4.28 (0.96)	4.38 (0.87)	5.827	<0.001
	Test 1	4.21 (0.86)	4.41 (0.80)	7.989	<0.001	Test 1	4.25 (0.85)	4.44 (0.74)	5.070	<0.001
	Test 2	4.26 (0.84)	4.37 (0.82)	4.707	<0.001	Test 2	4.28 (0.82)	4.46 (0.75)	5.137	<0.001
	Test 3	4.19 (0.86)	4.38 (0.80)	7.571	<0.001	Test 3	4.27 (0.84)	4.40 (0.78)	3.448	0.001
	Test 4	4.20 (0.88)	4.38 (0.79)	7.367	<0.001	Test 4	4.31 (0.83)	4.41 (0.76)	2.700	0.007
						Test 5	4.28 (0.82)	4.42 (0.78)	4.093	<0.001
						Test 6	4.31 (0.82)	4.44 (0.75)	3.797	<0.001
Total	Total	4.21 (0.86)	4.39 (0.80)	13.822	<0.001	Total	4.28 (0.83)	4.43 (0.76)	9.844	<0.001
	Test 1	4.25 (0.78)	4.42 (0.73)	7.294	<0.001	Test 1	4.05 (0.88)	4.19 (0.81)	3.395	0.001
	Test 2	4.29 (0.77)	4.40 (0.74)	4.848	<0.001	Test 2	4.06 (0.84)	4.19 (0.83)	3.633	<0.001
	Test 3	4.22 (0.79)	4.40 (0.73)	7.783	<0.001	Test 3	4.05 (0.85)	4.12 (0.84)	2.080	0.038
	Test 4	4.24 (0.79)	4.38 (0.75)	5.947	<0.001	Test 4	4.08 (0.87)	4.13 (0.83)	1.235	0.217
						Test 5	4.06 (0.86)	4.15 (0.84)	2.332	0.020
						Test 6	4.10 (0.83)	4.16 (0.81)	1.787	0.074
	Total	4.25 (0.78)	4.40 (0.74)	12.940	<0.001	Total	4.07 (0.85)	4.16 (0.83)	5.854	<0.001

*p < 0.05, **p < 0.01, ***p < 0.001.

#### 3.2.2. The correlation analysis between personal relationships and science achievement

PRAS scores were significantly and positively correlated with science achievement in Grade 4 (*r* = 0.180, *p* < 0.01) and Grade 8 (*r* = 0.159, *p* < 0.01). To further explore the correlation between student personal relationships and science achievement, we analyzed the data according to the test version, gender, and grade. We found that the correlation was roughly consistent and stable across these dimensions. See Table 8 for details.

**Table 8:** Correlations between personal relationships and science scores.

Relationship	Grade 4				Grade 8			
		Boys	Girls	Total		Boys	Girls	Total
Student-parent	Test 1	0.216**	0.103**	0.163**	Test 1	0.164**	0.164**	0.164**
	Test 2	0.161**	0.108**	0.135**	Test 2	0.095**	0.176**	0.133**
	Test 3	0.120**	0.119**	0.116**	Test 3	0.100**	0.105**	0.103**
	Test 4	0.118**	0.124**	0.117**	Test 4	0.084**	0.096**	0.089**
					Test 5	0.071*	0.107**	0.087**
					Test 6	0.090**	0.127**	0.106**
	Total	0.149**	0.110**	0.128**	Total	0.095**	0.111**	0.102**
Student-teacher	Test 1	0.197**	0.156**	0.177**	Test 1	0.203**	0.230**	0.215**
	Test 2	0.197**	0.189**	0.190**	Test 2	0.177**	0.163**	0.173**
	Test 3	0.154**	0.126**	0.136**	Test 3	0.173**	0.212**	0.191**
	Test 4	0.178**	0.144**	0.157**	Test 4	0.184**	0.139**	0.165**
					Test 5	0.188**	0.193**	0.189**
					Test 6	0.207**	0.113**	0.170**
	Total	0.172**	0.149**	0.158**	Total	0.180**	0.155**	0.170**
Student-peer	Test 1	0.262**	0.197**	0.230**	Test 1	0.217**	0.211**	0.213**
	Test 2	0.240**	0.226**	0.231**	Test 2	0.246**	0.220**	0.238**
	Test 3	0.198**	0.233**	0.207**	Test 3	0.195**	0.191**	0.196**
	Test 4	0.216**	0.184**	0.194**	Test 4	0.234**	0.166**	0.206**
					Test 5	0.208**	0.191**	0.198**
					Test 6	0.255**	0.164**	0.218**
	Total	0.221**	0.199**	0.206**	Total	0.216**	0.168**	0.196**
Total	Test 1	0.257**	0.176**	0.218**	Test 1	0.205**	0.209**	0.206**
	Test 2	0.223**	0.189**	0.204**	Test 2	0.182**	0.187**	0.187**
	Test 3	0.179**	0.167**	0.167**	Test 3	0.185**	0.162**	0.176**
	Test 4	0.176**	0.164**	0.164**	Test 4	0.165**	0.148**	0.158**
					Test 5	0.138**	0.160**	0.147**
					Test 6	0.176**	0.149**	0.164**
	Total	0.200**	0.167**	0.180**	Total	0.167**	0.148**	0.159**

*p < 0.05, **p < 0.01.

For boys in Grade 4, the correlation coefficient between the student-parent PRAS score and science achievement was 0.149 (*p* < 0.01) and those for each version of the science test ranged from 0.118 to 0.216 (all *p* < 0.01). For girls in Grade 4, the correlation coefficient was 0.110 (*p* < 0.01; ranging from 0.103 to 0.124 for each test version, all *p* < 0.01). In Grade 8, the correlation coefficient was 0.095 for boys (*p* < 0.01; ranging from 0.071 to 0.164, all *p* < 0.01) and 0.111 for girls (*p* < 0.01; ranging from 0.096 to 0.176, all *p* < 0.01).

In Grade 4, the correlation coefficient between student-teacher PRAS score and science achievement was 0.172 for boys (*p* < 0.01; ranging from 0.154 to 0.197, all *p* < 0.01) and 0.149 for girls (*p* < 0.01; ranging from 0.126 to 0.189, all *p* < 0.01). In Grade 8, it was 0.180 for boys (*p* < 0.01; ranging from 0.173 to 0.207, all *p* < 0.01) and 0.155 for girls (*p* < 0.01; ranging from 0.113 to 0.212, all *p* < 0.01).

In Grade 4, the correlation coefficient between student-peer PRAS scores and science achievement was 0.221 for boys (*p* < 0.01; ranging from 0.198 to 0.262, all *p* < 0.01) and 0.199 for girls (*p* < 0.01; ranging from 0.184 to 0.233, all *p* < 0.01). In Grade 8, it was 0.216 for boys (*p* < 0.01; ranging from 0.195 to 0.255, all *p* < 0.01) and 0.168 for girls (*p* < 0.01; ranging from 0.164 to 0.220, all *p* < 0.01).

In Grade 4, the correlation coefficient between the overall PRAS score and science achievement was 0.200 for boys (*p* < 0.01; ranging from 0.176 to 0.257, all *p* < 0.01) and 0.167 for girls (*p* < 0.01; ranging from 0.164 to 0.189, *p* < 0.01). In Grade 8, it was 0.167 for boys (*p* < 0.01; ranging from 0.138 to 0.205, all *p* < 0.01) and 0.148 for girls (*p* < 0.01; ranging from 0.148 to 0.209, all *p* < 0.01).

Correlation data were analyzed using the same repeated-measures General Linear Model as in Study 1 (see Table 9). We again found significant differences between personal relationships and achievement, which depended on the type of relationships (*p* < 0.001). Based on Table 8, we can see that among the three relationship types, the student-peer relationships were most closely related to science achievement.

**Table 9:** Results of the intra-group factor test.

Source	*SS*	*df*	*MS*	*F*	*P*
Personal relationships	0.077	2	0.039	72.767	<0.001
Gender	0.001	2	0.001	1.075	0.353
Grade	0.003	2	0.001	2.692	0.083
Gender × Grade	0.004	2	0.002	4.193	0.024
Error(personal relationships)	0.017	32	0.001		

*p < 0.05, **p < 0.01, ***p < 0.001, Bonferroni-corrected.

#### 3.2.3. Multivariate ANOVA

Multi-factor ANOVAs were used to investigate gender and grade differences in the relationship between student personal relationships and science achievement. We found no significant grade or gender differences in the correlations between the three relationship types and science achievement (*p* > 0.05, Bonferroni-corrected). See Table 10 for details.

**Table 10:** ANOVA for gender and grade in relation to personal relationships and achievement.

	Student-parent relationship					Student-teacher relationship				Student-peer relationship			
	*F*	*df*	*P*	*η^2^ p*		*F*	*df*	*p*	*η^2^ p*	*F*	*df*	*p*	*η^2^ p*
Gender	0.151	1	0.702	0.009		2.302	1	0.149	0.126	6.138	1	0.025	0.277
Grade	1.534	1	0.233	0.087		1.084	1	0.313	0.063	1.068	1	0.317	0.063
Gender × Grade	5.177	1	0.037	0.244		0.266	1	0.613	0.016	0.555	1	0.467	0.034
Error		16					16				16		

*p < 0.05, **p < 0.01, ***p < 0.001, Bonferroni-corrected.

### 3.3. Discussion

The results of Study 2 support our hypothesis the conclusion of Study 1; student-peer relationships had the closest association with academic achievement, compared with student-parent and student-teacher relationships. In Study 2, we used science performance as the index of academic performance to verify the relationships we found in Study 1.

Results of the correlation analysis confirmed a significant positive correlation between personal relationships and academic performance, with the student-peer relationships being the most strongly associated academic performance, in both Grade 4 and Grade 8 and among both boys and girls, even after changing the discipline categories.

However, we found that the correlation between student-peer relationships and science achievement did not depend on grade, which was inconsistent with the results from Study 1 in which the same relationship did depend on grade when the subject matter was mathematics. Thus, performance in different disciplines (math vs. science) might not be affected by social relationships in the same way. We speculate that the differences between math and science could be related to the extent that the students care, the time spent studying, and the point-weight of the exam. In China, because math is a subject used for school entrance examinations, it is considered important in every grade. Due to the constant external factors in math learning, we speculate that the grade difference in the correlation between the student-peer relationships and math achievement (Study 1) is simply related to age-related developmental differences, such as cognition. In contrast, it is true that neither students, teachers, or parents pay much attention or invest much in science education in primary schools, as science is not part of any entrance exam. However, science does receive more attention in junior high school as it begins to become incorporated in entrance exams. According to the report from the Program for International Student Assessment (Schleicher, 2019), the average time that Chinese students spent on math and science courses were about 5 h per day, each. We speculate that the change in external factors that affect science learning eclipsed age-related developmental differences, resulting in no significant grade difference in the correlation between the student-peer relationships and science achievement.

## 4. General discussion

In 2018 and 2019, a large sample of primary and middle school students were selected as research participants, and different disciplines were used to explore the relationship between three types of personal relationships and academic performance. The results from Study 1 and Study 2 strongly support our hypothesis that among the three types of important personal relationships (student-parent, student-teacher, and student-peer), the student-peer relationships were the most closely related to academic performance.

### 4.1. The significant positive correlation between personal relationships and academic achievement

The positive correlation between personal relationships and academic performance in this study is consistent with most previous studies. According to the self-determination theory (SDT), supportive relationships may fulfill students basic psychological need for social relatedness (Deci and Ryan, 2000). When this need is met, adolescents feel connected to their teachers and peers, which fosters their motivation to behave in socially appropriate ways and concentrate on learning. Kiuru et al. (2014) also found that student academic performance can be promoted by increasing the support students receive from peers, parents, and teachers because such increased support leads to better task focus when learning. According to Soe (2020), the relationship between social support that students received from their parents, teachers, and peers and academic achievement were significantly correlated with each other; the more students get social support, the better their academic performance.

### 4.2. The student-peer relationships were highly related to academic achievement

This study confirmed our hypothesis that student-peer relationships would have a closer association with academic achievement than student-parent or student-teacher relationships. This result is consistent with previous studies. Kindermann (2016) found that students interactions with agemates enhanced their learning over and above the provisions of adult educators; many children appear to go to school or to like school (better) because of their peers and friends. Peer acceptance can have motivational benefits that enhance the learning process; children who enjoy positive relationships with peers also tend to be more engaged in academic tasks and even excel at academic tasks more than those who have problems in their peer relationships (Wentzel, 2017; Wentzel et al., 2020). Adolescents who are victimized by their peers are less motivated to attend school and may miss learning opportunities (Eisenberg et al., 2003).

The current study strongly supports this view. The item on the PRAS that correlated most with performance (highest overall correlation, −0.185 in Study 1; fourth highest overall correlation, −0.156 in Study 2) was, “It is difficult for me to participate in the discussions and activities of my classmates.” The PRAS question that correlated second-most overall with achievement scores (−0.181) was an item from Study 2, “I want to participate in discussions and activities with my class.” See Supplementary Materials for details. Thus, successful participation in discussions and activities with their peers is closely related to students’ academic performance. We propose that in a safe and effective learning environment created by good peer relationships, students are more willing to participate in activities, and the higher the frequency of their collaborative interactions, the better their academic performance.

This conclusion verifies the Social Impact Theory (SIT). The strength (S), the immediacy (I), and the number (N), as proposed by SIT, is further discussed below based on the results of this study, existing studies, and the current situation of education.

First, compared with other groups, peers have more influence on students. Strength (S) is strongly supported by the high correlation between academic performance and peer relationships. As children move from primary school to middle school, the social support function from parents, teachers, and peers seems to change, and by middle and late childhood, close friendships become an important social support system for children. Furman and Buhrmester (1992) compared with parents, school-aged students are more willing to rely on friends to encourage and support them in coping with academic pressure.

Second, in the context of learning, students naturally have more direct contact and closer relationships with their peers than with others. Immediacy (I) is strongly supported by the high correlation between academic performance and peer relationships. In the famous Chinese story “The three times moving of Meng Ke’s mother “, Meng Ke’s mother chose good environments and companions for the child and moves many times. Known since ancient times, people have paid attention to the effect that companions have on their children.

Finally, peers are the people with whom students have the most contact. The Number (N) again is strongly supported by the high correlation between academic performance and peer relationships. Most researchers agree that the number of partners affects an individual’s performance. For example, Carter and Hughes (2005) found that students with disabilities had higher levels of social interaction and contact with the general curriculum when they worked with two peers than when they worked with one.

In addition, we believe that cooperative inquiry teaching, which has been advocated by educators in recent years, has a positive effect on peer closeness. As early as 2001, China mentioned and advocated cooperative learning in the Decision of the State Council on the Reform and Development of Basic Education, pointing out that it encourages cooperative learning, promotes mutual communication and development among students, and promotes teachers and students to learn from each other. The Outline of Basic Education Curriculum Reform (Trial), issued in the same year, once again proposed that comprehensive practical activities should be taken as compulsory courses from primary school to senior high school. Research-based learning is a key focus of this new plan, implemented in order to develop communication and cooperation among students.

In the following years, cooperative learning was discussed as China’s education and teaching reform deepened. Most teachers are actively practicing and guiding students to cooperate in learning. A series of studies confirmed the effect of peer cooperative learning. For instance, Veldman et al. (2020) found that cooperative learning may lead to improved group work behavior in young pupils (6–7 years old). Molla and Muche (2018) showed that a significant gain in learning occurred *via* a cooperative learning-achievement division followed by a cooperative discussion group. We speculate that in efficient cooperative learning processes, students and peers become closer and the importance of peers in a student’s learning increases.

### 4.3. Educational implications

#### 4.3.1. The importance of participation and cooperation

The results of this study remind educators that in addition to cognitive factors, personal relationships, especially peer relationships, need to be considered with regards to student academic performance. Student interactions with their peers in discussions or activities is closely related to academic performance. This enlightens our daily teaching work: (1) Teachers should guide students to integrate into the collective learning process, create learning opportunities through communication, encourage students to participate in discussions and activities, help them learn how to cooperate, and should not deprive students of time and space for independent communication; (2) teachers should also create a class and campus atmosphere of unity, mutual assistance, and friendship, guide students with a positive learning attitude to inspire others around them, and should not cultivate antagonistic emotions or over-emphasize competition; (3) teachers should also strive to improve the environmental conditions and adjust the space between students (such as seat adjustment) to promote peer relationships and increase the possibility of discussion and exchange. A series of studies have shown that learning spaces are becoming an important fulcrum of school reform. Flexible learning spaces have a significant impact on learning outcomes, including improving academic performance, promoting teacher-student interactions in class, and improving the learning experience (Brooks and Baepler, 2012; Baepler and Walker, 2014; Gremmen et al., 2018).

#### 4.3.2. The importance of school and class construction

The results provided some evidences for the valuable of the existence of schools and classrooms. In recent years, many schools are exploring the abolition of classroom-teaching systems. Other are considering improving the classroom-teaching system by using an “Optional Class System” or other teaching methods. “With the rise of intelligent technology, the widespread availability of free learning opportunities signals the decline of existing curriculum structures and the collapse of school systems, the disappearance of traditional teaching staff, and the emergence of individuals as producers and practitioners of knowledge” (Organization for Economic Cooperation and Development, 2020). The natural limitations of traditional school education and classroom-teaching systems (such as unified teaching requirements and methods) make it difficult to meet the diverse learning needs of individuals.

However, the results of this study also remind us to think again. Collective teaching is not without advantages, and classrooms are also necessary. We believe that although ubiquitous learning resources are everywhere, the physical learning space is still irreplaceable. The process of knowledge acquisition is bets when it is not a solitary endeavor. It can be affected by many social factors; peer interactions and the quality of peer relationships within physical learning spaces are closely related to academic performance. Our view is supported by the report by the United Nations Educational, Scientific, and Cultural Organization, which states in 2015 that “learning should not be an individual matter, but a social experience that requires learning with others and through discussion and debate with peers and teachers.” The classroom structure provides an environment for students to promote and interact with their peers as a collective, where they study together and gain a sense of belonging and security. It provides stable personal support and assists with learning, which is difficult to establish in an “Optional Class System.” We know that in the “Optional Class System,” choosing courses according to interest and level can give more space to a group of students with strong autonomy and good learning foundation, but for other students, it might bring about confusion. This could lead to the strong becoming stronger and the weak becoming weaker (Schofield, 2010; Hamilton and O’Hara, 2011; Wilkinson et al., 2015; Smyth, 2016).

Based on this, we believe that schools and classrooms remain valuable and should continue to exist. In the future, the construction of schools and classrooms needs to be constantly improved, which will be through a process that connects the past to the future.

### 4.4. Limitations of the study and implications for future research

To the best of our knowledge, this is the first analysis that has compared how well three important types of personal relationships correlate with academic performance. Therefore, more research is needed to confirm our conclusion that student-peer relationships are the most important. Additionally, some limitations need to be noted.

First, this study only divided personal relationships into three types, but did not continue to refine the indicators. According to existing studies, student-parent, student-teacher, and student-peer relationships are defined in various ways and classified by indicators (for example, student-peer relationships are refined into peer acceptance and peer rejection (Zhang et al., 2013)). Future studies can consider further refining the three dimensions we used here.

Second, although we designed two studies to confirm our results, the analyses were correlational; therefore, causal inferences cannot be drawn. Little is known about how the student-peer relationships as a variable affects academic performance and the underlying processes that can explain these relationships. The same goes for our speculation that cooperative learning is closely related to our conclusion. It is necessary to carefully consider how learning style affects student relationships and academic performance, and what variables should be controlled for further research on this scientific issue.

Third, this study selected Grade 4 and Grade 8 students in Qingdao, Shandong Province, China as representatives for the investigation. In order to make the research results more representative, future research should focus on students from more grade levels with more varied demographic profiles. This might reveal a pattern of findings and developmental characteristics not captured by the current study. Here, we have discussed the correlation between personal relationships and academic performance in math and science. In the next step, other disciplines should be considered to enrich the existing research results. Furthermore, the samples of this study were only from China, where interpersonal relationships were of particular importance, and the representativeness has certain limitations. Future studies could be extended to more diverse samples to investigate the correlation between interpersonal relationships and academic performance.

## 5. Conclusion

Through the investigation and analysis of a large data sample, we have compared how well three important personal relationships (student-parent, student-teacher, and student-peer) correlate with academic performance. We found that the student-peer relationships were most closely related to academic performance. The conclusion of this study helps us to further understand the relationship between personal relationships and academic performance. At the same time, it also reminds educators to pay attention to the personal relationships among their students, especially the peer relationships. It also reminds educators to create opportunities and environmental conditions for exchange and learning, encourage discussion and cooperation, and create a united classroom and campus atmosphere.

## Data availability statement

The raw data supporting the conclusions of this article will be made available by the authors, without undue reservation.

## Ethics statement

The studies involving human participants were reviewed and approved by Experimental Ethics Committee of basic teaching center of Ocean University of China. Written informed consent to participate in this study was provided by the participants’ legal guardian/next of kin.

## Author contributions

XY, HW and XZ designed experiments and revised the manuscript. XY and HW collected data and analyzed data. XW, HZ, XZ and MS analyzed data and wrote the manuscript. All authors contributed to the article and approved the submitted version.

## Funding

This work was supported by the MOE (Ministry of Education of the People’s Republic of China) Project of Humanities and Social Sciences [No. 18YJC190030] and Qingdao Municipal Planning Project of Social Sciences [No. QDSKL2001017].

## Conflict of interest

The authors declare that the research was conducted in the absence of any commercial or financial relationships that could be construed as a potential conflict of interest.

## Publisher’s note

All claims expressed in this article are solely those of the authors and do not necessarily represent those of their affiliated organizations, or those of the publisher, the editors and the reviewers. Any product that may be evaluated in this article, or claim that may be made by its manufacturer, is not guaranteed or endorsed by the publisher.

## Supplementary material

The Supplementary material for this article can be found online at: https://www.frontiersin.org/articles/10.3389/fpsyg.2023.1012701/full#supplementary-material
