# Supplementary material for: Academic achievement is more closely associated with student-peer relationships than with student-parent relationships or student-teacher relationships
Source: Front Psychol. 2023 Feb 16;14:1012701. doi: 10.3389/fpsyg.2023.1012701 (PMC9978389; doi:10.3389/fpsyg.2023.1012701)
Supplement: Supplementary file 1 [file Data_Sheet_1.docx]

# Supplemental Materials for

**Student Relationships with Peers are Highly Associated with Academic Achievement**

## The correlation between each question of PRAS and math scores

The following table shows the correlation between scores for each PRAS question and math scores. A significant correlation was found between various dimensions of personal relationships and math achievement. For example, the correlation coefficient between scores for the question, “It is difficult for me to participate in the discussions and activities of my classmates” and math performance was −.207 (*P* < .01) for 4^th^ graders and −.185 (P < .01) for 8th graders. In another example, the correlation coefficient between the scores for the question, “Teachers treat me fairly” and their math performance was .121 (P < .01) for 4th graders and .126 (P < .01) for 8th graders. See Supplemental Table 1 for details.

## The correlation between each question of PRAS and science scores

The following table shows the correlation between scores for each PRAS question and science scores. A significant correlation was found between various dimensions of personal relationships and science achievement. For example, the correlation coefficient between scores for the question, “My parents don’t give me moral support” and science performance was −.170 (P < .01) for 4^th^ graders and −.129 (P < .01) for 8^th^ graders. In another example, the correlation coefficient between scores for the question, “I want to participate in discussions and activities with my class” and science performance was .145 (P < .01) for 4^th^ graders and .181 (*P* < .01) for 8^th^ graders. See Supplemental Table 2 for details.

**Supplemental Table 1**

Correlation between the score for each PRAS question and math scores

| Relationship | Grade 4 | Grade 8 | Item |
| --- | --- | --- | --- |
| Student-parent | .092** | .056** | 1. I feel very happy with my parents. |
|  | .092** | .088** | 2. My parents listen to me carefully. |
|  | -.132** | -.122** | 3. My parents lack confidence and spiritual support for me. |
|  | .104** | .070** | 4. My parents will often give me support and encouragement. |
| Student-teacher | .134** | .112** | 5. When I’m in trouble, teacher help me in time. |
|  | .121** | .126** | 6. Teachers treat me fairly. |
|  | .125** | .113** | 7. My teachers like me very much. |
| Student-peer | -.207** | -.185** | 8. It is difficult for me to participate in the discussions and activities of my classmates. |
|  | .092** | .102** | 9. When I have difficulty, my classmates care about me and help me. |
|  | -.179** | -.142** | 10. I have been bullied by my classmates in school. |

*Note.* **p* < .05, ***p* < .01

**Supplemental Table 2**

Correlation between the score for each question of the PRAS and science scores

| Relationship | Grade 4 | Grade 8 | Item |
| --- | --- | --- | --- |
| Student-parent | .114** | .057** | 1. I feel very happy with my parents. |
|  | .113** | .090** | 2. My parents will encourage me. |
|  | -.099** | -.061** | 3. I have disagreements or quarrel with my parents. |
|  | -.170** | -.129** | 4. My parents don’t give me moral support. |
|  | .048** | .059** | 5. My parents do interesting things with me. |
|  | -.079** | -.091** | 6. My parents force me to do things I don't like to do. |
|  | .049** | .058** | 7. I can share my heart's secrets and feelings with my parents. |
|  | .042** | .094** | 8. My parents can understand me. |
| Student-teacher | .105** | .110** | 9. When I’m in trouble, teacher help me in time. |
|  | .146** | .162** | 10. Teachers treat me fairly. |
|  | -.140** | -.134** | 11. Teachers are not friendly to me. |
|  | .104** | .161** | 12. I like my teachers very much. |
| Student-peer | .145** | .181** | 13. I want to participate in discussions and activities with my class. |
|  | -.182** | -.156** | 14. It is difficult for me to participate in the discussions and activities of my classmates. |
|  | .090** | .132** | 15. When I have difficulty, my classmates care about me and help me. |
|  | -.142** | -.142** | 16. I have been bullied by my classmates in school. |
|  | .130** | .120** | 17. I have good times with my classmates. |

*Note.* **p* < .05, ***p* < .01.
